# Tartaric acid-branched polyethyleneimine carbon dots promote repair of bone defect via osteogenic differentiation

**DOI:** 10.1093/rb/rbaf030

**Published:** 2025-05-16

**Authors:** Soon Chul Heo, Hae Won Shin, Dong Joon Lee, Franklin Garcia-Godoy, Bo Ram Keum, Yong Hoon Kwon, Hyung Joon Kim

**Affiliations:** Department of Oral Physiology, Periodontal Diseases Signaling Network Research Center, Dental and Life Science Institute, School of Dentistry, Pusan National University, Yangsan 50612, Republic of Korea; Institute of Tissue Regeneration Engineering (ITREN), Mechanobiology Dental Medicine Research Center, Dankook University, Cheonan 31116, Republic of Korea; Department of Neurology, College of Medicine, University of Tennessee Health Science Center, Memphis, TN 38163, USA; Oral and Craniofacial Health Sciences, Adams School of Dentistry, University of North Carolina at Chapel Hill, Chapel Hill, NC 27514, USA; Department of Bioscience Research, College of Dentistry, University of Tennessee Health Science Center, Memphis, TN 38103, USA; Department of Oral Physiology, Periodontal Diseases Signaling Network Research Center, Dental and Life Science Institute, School of Dentistry, Pusan National University, Yangsan 50612, Republic of Korea; Department of Dental Materials, School of Dentistry, Pusan National University, Yangsan 50612, Republic of Korea; Department of Oral Physiology, Periodontal Diseases Signaling Network Research Center, Dental and Life Science Institute, School of Dentistry, Pusan National University, Yangsan 50612, Republic of Korea

**Keywords:** carbon dots, tartaric acid, branched polyethyleneimine, osteogenesis, bone repair

## Abstract

Treating bone defects is a critical challenge in regenerative medicine. Carbon nanomaterials, with their unique physicochemical properties, offer significant potential for enhancing bone regeneration. In this study, we developed tartaric acid (TA)-based carbon dots (CDs) by synthesizing TA with branched polyethyleneimine (bPEI). These TA-bPEI CDs were systematically evaluated to determine their effects on osteogenic differentiation in human bone marrow-derived mesenchymal stem cells (BMSCs) and their capacity to repair calvarial defects in an *in vivo* model. Characterization of TA-bPEI CDs revealed a size of approximately 10 nm and a positive surface charge. The CDs exhibited fluorescence emission peaks between 464 and 506 nm under excitation wavelengths of 340–440 nm. Cytotoxicity assays demonstrated that TA-bPEI CDs maintained BMSC viability at concentrations up to 250 μg/ml. However, at concentrations of 500 μg/ml and above, apoptosis was induced. Treatment with TA-bPEI significantly enhanced osteogenic differentiation *in vitro*, as evidenced by increased expression of osteogenic-specific proteins such as Runx2, ALP, OCN and OPN. *In vivo*, the application of TA-bPEI CDs in a mouse calvarial defect model promoted robust new bone formation, reduced defect gaps, and improved bone morphometric parameters, including bone volume fraction and trabecular thickness. These results suggest that TA-bPEI CDs enhance osteogenesis by directly stimulating osteogenic differentiation and upregulating osteogenesis-specific genes. This study demonstrates the high potential of TA-bPEI CDs as a novel nanomaterial for bone regeneration applications.

## Graphical abstract

**Figure:**
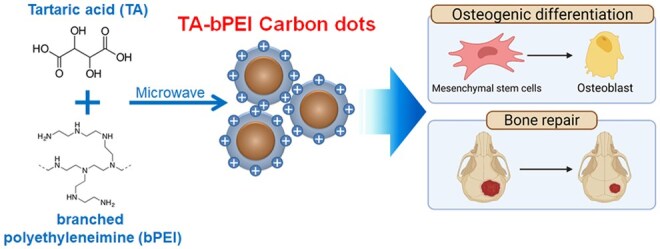

## Introduction

Bone defect repair is a complex and continuous process influenced by many factors such as hormones, proteins, growth factors and osteogenic stem cells [1]. Managing significant fractures and bone defects poses a considerable challenge in orthopedic surgery. Many investigators are currently focusing on the integration of growth factors and other compounds with remarkable biological activities into scaffold biomaterials to enhance the healing of bone deformities. Treatment of bone defects involves the use of hydrogels [2], fiber spinning [3] and carbon-based materials [4].

Carbon (quantum) dots (CDs) are carbon-based nanosized dots that are generally smaller than 10 nm. These new nanoparticles can be obtained by many different methods via heating or chemical processes using carbon-containing chemicals or various natural resources [5, 6]. Although they are carbon-based, CDs also contain O, N and other elements, such as P, S, B, etc, depending on the chemicals used for synthesis [7, 8]. CDs have a variety of applications in many fields, ranging from environmental sciences to life sciences. Active research in medicine is due to its excellent applicability in imaging, sensing and treating bacteria and systemic diseases, in conjunction with CD-based drug delivery and photon/ultrasound-mediated catalytic and/or dynamic therapies [9, 10]. Additionally, some CDs have been reported to promote osteogenic differentiation [11, 12]. CDs can be used as carriers independently or in combination with other agents, depending on the application. In many cases, CDs are passivated by negatively charged –OH and –COOH functional groups derived from the main carbon source, such as citric acid or ascorbic acid, thereby resulting in a negative zeta potential [13–15]. (Linear, Branched) polyethyleneimine (PEI) is an organic polyamine polymer. One nitrogen atom in every three atoms in the PEI backbone confers positive charges through the protonation of amino nitrogen. Cationic PEI can form complexes with negatively charged CDs through electrostatic interactions and bind to the negatively charged outer surfaces of cells. PEI is a widely studied transfection reagent; however, if used in excess, toxicity can be caused by the disruption of the cell and mitochondrial membrane. According to the studies, high molecular weight PEI, such as branched PEI of 1800 Da, demonstrates negligible transfection efficiency but much lower cytotoxicity than other frequently used PEI of 25 kDa [16, 17].

Recent breakthroughs in tissue engineering have highlighted the potential of mesenchymal stem cells (MSCs) as alternatives to established techniques. These advancements are linked to reduced morbidity, decreased complication rates and enhanced functionality in bone regenerative therapies [18, 19]. Various tissues serve as sources of MSCs, which exhibit characteristics such as self-renewal and clonogenic ability and possess the capacity to develop into bone, cartilage and adipose tissues [20, 21]. Among various sources, MSCs obtained from bone marrow (BMSCs) have attracted considerable interest in bone regeneration research [22].

In the present study, tartaric acid (TA) was combined with branched polyethyleneimine (bPEI) and heated using a microwave. The resultant TA-bPEI CDs were characterized and tested using BMSC at low concentrations to explore whether positively charged low-concentration TA-bPEI CDs potentiate osteogenic differentiation and promote the healing of *in vivo* bone defects, which are essential for determining whether the tested CDs are applicable for bone regeneration.

## Materials and methods

### Materials

Alpha-modified minimum essential medium (α-MEM) was obtained from Welgene, Inc. (Daegu, South Korea). Gibco (Thermo Fisher Scientific Inc., Waltham, MA, USA) supplied fetal bovine serum (FBS), trypsin-EDTA and a penicillin–streptomycin mixture. Antibodies specific to Runx2, OPN, OCN and β-actin were sourced from Cell Signaling Technology (Danvers, MA, USA).

### Synthesis of TA-bPEI CDs

For the synthesis of TA-bPEI CDs, TA (500 mg) was completely dissolved in 20 ml of deionized water in a glass beaker (Beaker A). Separately, 200 mg of branched PEI (bPEI; MW: 1800 Da) was fully dispersed in 10 ml of deionized water within another beaker (Beaker B). The contents of both beakers were then combined, stirred for 10 min and subjected to sonication at room temperature for another 10 min to ensure homogeneity. The resulting mixture was exposed to microwave irradiation at 600 W for 5 min. Once cooled to ambient temperature, an additional 20 ml of deionized water was added to aid in dissolution. The resulting brown solution underwent centrifugation at 15 000 rpm for 10 min to remove larger aggregates, then was filtered using a 0.22 μm membrane. The supernatant underwent dialysis (MWCO = 3.5 kDa) in deionized water for 24 h. Ultimately, the water-dispersible TA-bPEI CDs were acquired through vacuum drying. For comparative analysis, TA CDs were synthesized by placing TA powder in a covered crucible and heating it in a muffle furnace at 280°C for 2 h with a temperature increase of 10°C/min, then allowing it to cool naturally. The cooled sample was finely ground using a mortar, dispersed in distilled water, centrifuged at 15 000 rpm for 10 min and passed through a 0.22 μm membrane filter. The final TA CDs were obtained after vacuum-drying the supernatant.

### Characterization of the synthesized CDs

The structural characteristics of the produced CDs were analysed using high-resolution transmission electron microscopy (HRTEM) with a TALOS F200X system (Thermo Fisher Scientific, Waltham, MA, USA). The surface charge, expressed as zeta potential, was assessed using a Malvern Zetasizer Nano ZSP. The elemental composition was examined using X-ray photoelectron spectroscopy (XPS) with a JB Scientific AXIS SUPRA spectrometer. Fourier-transform infrared (FT-IR) spectroscopy was used to identify the functional groups in the samples with a Thermo Scientific IS50 spectrometer. The crystalline structure of the CDs was determined by X-ray diffraction (XRD) analysis performed on an X'Pert3 Powder diffractometer (Malvern Panalytical). To assess optical properties, UV–visible (UV–vis) absorption spectra were measured using a JASCO V-670 spectrophotometer, while photoluminescence (PL) spectra were recorded with a Cary Eclipse spectrophotometer (Agilent Technologies, Santa Clara, CA, USA). Additionally, fluorescence characteristics of CDs at the single-cell level were analysed through the lambda-scan function of an LSM 700 confocal microscope (Carl Zeiss, GmbH), where specimens were excited with a 405 nm laser, and emission data were collected in 15-nm increments between 420 and 630 nm. The mean fluorescence intensity of regions containing CDs was quantified using ImageJ software.

### Cell culture

Human BMSCs were sourced from ScienCell (Carlsbad, CA, USA) and cultured in α-MEM with 10% fetal bovine serum (FBS) under a humidified 5% CO_2_ environment at 37°C. Upon reaching 90% confluence, the cells were subcultured, with only those passaged fewer than seven times used in the experiments.

### Flow cytometry

BMSCs were exposed to varying concentrations of either control bPEI or TA-bPEI for 48 h, after which they were detached using trypsin/EDTA. The harvested cells were resuspended in binding buffer and labeled with 5 μl of FITC-annexin V and 5 μl of propidium iodide (PI) using the FITC Annexin V Apoptosis Detection Kit I (BD Pharmingen). Following a 15-min incubation at 25°C in the dark, fluorescence-labeled cells were examined using a FACS Canto II flow cytometer, and data were acquired with DIVA software (Becton Dickinson, USA).

### Cytotoxicity and cell viability assay

For the cytotoxicity assessment, BMSCs were plated in 96-well plates at a density of 5 × 10³ cells per well and cultured with or without different concentrations of CDs for 48 h. In proliferation assays, BMSCs were treated with either 1 µg/ml control bPEI or TA-bPEI for a duration of three days. At specified time intervals, cell viability was assessed by introducing 20 µl of Cell Counting Kit-8 (CCK-8) reagent (Dojindo, Rockville, MD, USA). The absorbance was then measured at 450 nm using an Opsys MR microplate reader (DYNEX Technologies Inc., Denkendorf, Germany).

### Osteoblast differentiation

Osteoblast differentiation was carried out following established protocols [23]. In summary, BMSCs were maintained in an osteogenic medium supplemented with 100 μg/ml ascorbic acid and 10 mM β-glycerophosphate, with or without 1 μg/ml TA-bPEI CDs, for four days, replacing the medium every other day. To evaluate differentiation, alkaline phosphatase (ALP) staining was conducted using an ALP assay kit (Sigma-Aldrich) in accordance with the manufacturer's instructions. Microscopic imaging of stained cells was performed with a Nikon Eclipse Ts2 microscope. ALP activity was measured using the 1-Step PNPP substrate solution (Thermo Fisher Scientific, Waltham, MA, USA) following the manufacturer's protocol, and absorbance was recorded at 405 nm.

### Reverse transcriptase-polymerase chain reaction

Total RNA was extracted using the RNeasy Mini Kit (Qiagen) following the manufacturer’s protocol. Then, 1 μg of RNA was reverse-transcribed under standard conditions using Superscript II (Invitrogen, Waltham, MA, USA). For quantitative PCR (qPCR), 50 ng of complementary DNA was mixed with SYBR Green PCR Master Mix (Applied Biosystems, Foster City, CA, USA) and amplified for 40 cycles using a QuantStudio system (Applied Biosystems). Each experiment was performed in triplicate, with β-actin used as the reference gene for normalization. The relative expression levels were calculated using the 2^–ΔΔ^^*Ct*^ method. The primer sequences used in this study were as follows: OPN, 5′-GCCAGCAACCGAAGTTTTCAC-3′, 5′-TGCACCATTCAACTCCTCGC-3′; Runx2, 5′-TGCTTTGGTCTTGAAATCACA-3′, 5′-TCTTAGAACAAATTCTGCCCTTT-3′; ALP, 5′-GCTGTAAGGACATCGCCTACCA-3′, 5′-CCTGGCTTTCTCGTCACTCTCA-3′; OCN, 5′-CAGCGAGGTAGTGAAGAGAC-3′, 5′-TGAAAGCCGATGTGGTCAG-3′; β-actin, 5′-ACTCTTCCAGCCTTCCTTCC-3′, 5′-TGTTGGCGTACAGGTCTTTG-3′.

### Western blotting

A RIPA buffer solution containing 50 mM Tris (pH 8.0), 150 mM NaCl, 0.5% sodium deoxycholate, 1 mM EGTA, 1% Triton X-100, 10 mM NaF, PMSF and a broad-spectrum protease inhibitor cocktail was used to extract cell lysates. A total of 20 μg of extracted protein was separated by SDS-PAGE using 10% or 15% gels. After electrophoresis, proteins were transferred to nitrocellulose membranes and blocked with 5% skim milk for 1 h. The membranes were then incubated overnight with primary antibodies, followed by a 1-h incubation with horseradish peroxidase-conjugated secondary antibodies. Protein detection was carried out using enhanced chemiluminescence reagents (Amersham Pharmacia Biotech Ltd). To ensure equal protein loading, β-actin was used as a reference control.

### Calvarial defect model

Animal care and experimental procedures complied with the Principles of Laboratory Animal Care and were performed under protocols approved by the Pusan National University Institutional Animal Use and Care Committee (PNU‐2023‐0271). Male C57BL/6J wild-type mice (6 weeks old, weighing 22–25 g) were obtained from Orient Co. Ltd (Gapyeong, Korea). The calvarial defect model was established as described previously [24]. Mice were anesthetized through intraperitoneal injection of 400 mg/kg 2,2,2-tribromoethanol (Avertin; Sigma-Aldrich, St Louis, MO, USA). A full-thickness incision was made in the scalp, followed by periosteal elevation to expose the calvarial bone. Bone defects were created using a 4 mm diameter hollow trephine bur and a hand drill. To minimize heat-induced damage during drilling, sterile saline was applied continuously. Immediately after defect formation, 20 μl of Matrigel (Corning, Glendale, AZ, USA) containing or lacking 1 μg/ml TA-bPEI was applied to the site. Twelve weeks post-surgery, mice were euthanized using CO_2_ inhalation, and the calvariae were harvested for micro-CT imaging and histological examination. Samples were fixed in 4% paraformaldehyde and analysed using a micro-CT system (InspeXio SMX-90 CT; Shimadzu Corp., Kyoto, Japan). Images were acquired through 360° rotations at 90 kV and 100 mA, then reconstructed using inspeXio SMX-90CT software (Shimadzu Corp., Kyoto, Japan). Bone structural parameters, such as bone volume fraction (BV/TV), trabecular thickness (Tb.Th) and trabecular separation (Tb.Sp), were assessed using ImageJ software. To determine the defect gap—the distance between the newly formed bone edges—three sequential H&E-stained sections were measured using ImageJ software. Additionally, new bone (NB) volume was quantified based on Masson’s trichrome staining images.

### Statistical analysis

Statistical comparisons between the two groups were performed using Student’s *t*-test. For multiple group analyses, one-way or two-way analysis of variance (ANOVA) followed by the Bonferroni *post hoc* test was used. A significance threshold of *P* < 0.05 was set for all analyses. Data are expressed as mean ± standard deviation (SD), with findings derived from a minimum of three independent experiments.

## Results

### Morphology and zeta potential of TA-bPEI CDs

**Figure 1.**
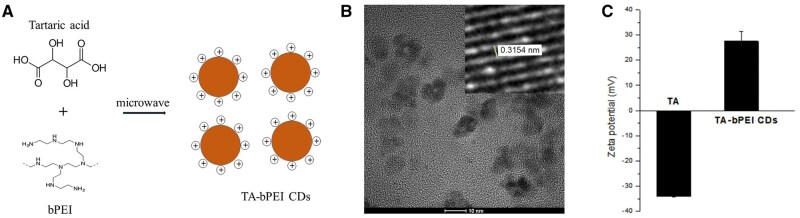
Characterization of TA-bPEI CDs. (**A**) Schematic representation of TA-bPEI CDs synthesized via a microwave-assisted reaction using tartaric acid and bPEI. (**B**) HRTEM image of TA-bPEI CDs and lattice spacing. (**C**) Zeta potential of TA-bPEI CDs. Negative zeta potential of TA changed to positive after combining with bPEI.

The morphologies of the synthesized CDs and their surface potentials are shown in Figure 1. The synthesis of the CDs using TA and bPEI is schematically illustrated in Figure 1A. TA was used as a carbon source, and bPEI endowed the CDs with positive charges. Figure 1B shows the morphology of the obtained CDs. According to high-resolution TEM (HRTEM), the dispersed CDs were of near to 10 nm size with approximately 0.32 nm lattice spacing of graphite, which corresponds to the (0, 0, 2) plane. Zeta potential of TA CDs by DLS at pH 7 was −34.1 ± 0.3 mV, changed to 27.5 ± 3.75 mV for TA-bPEI CDs (Figure 1C).

### XPS and FT-IR of TA-bPEI CDs

**Figure 2.**
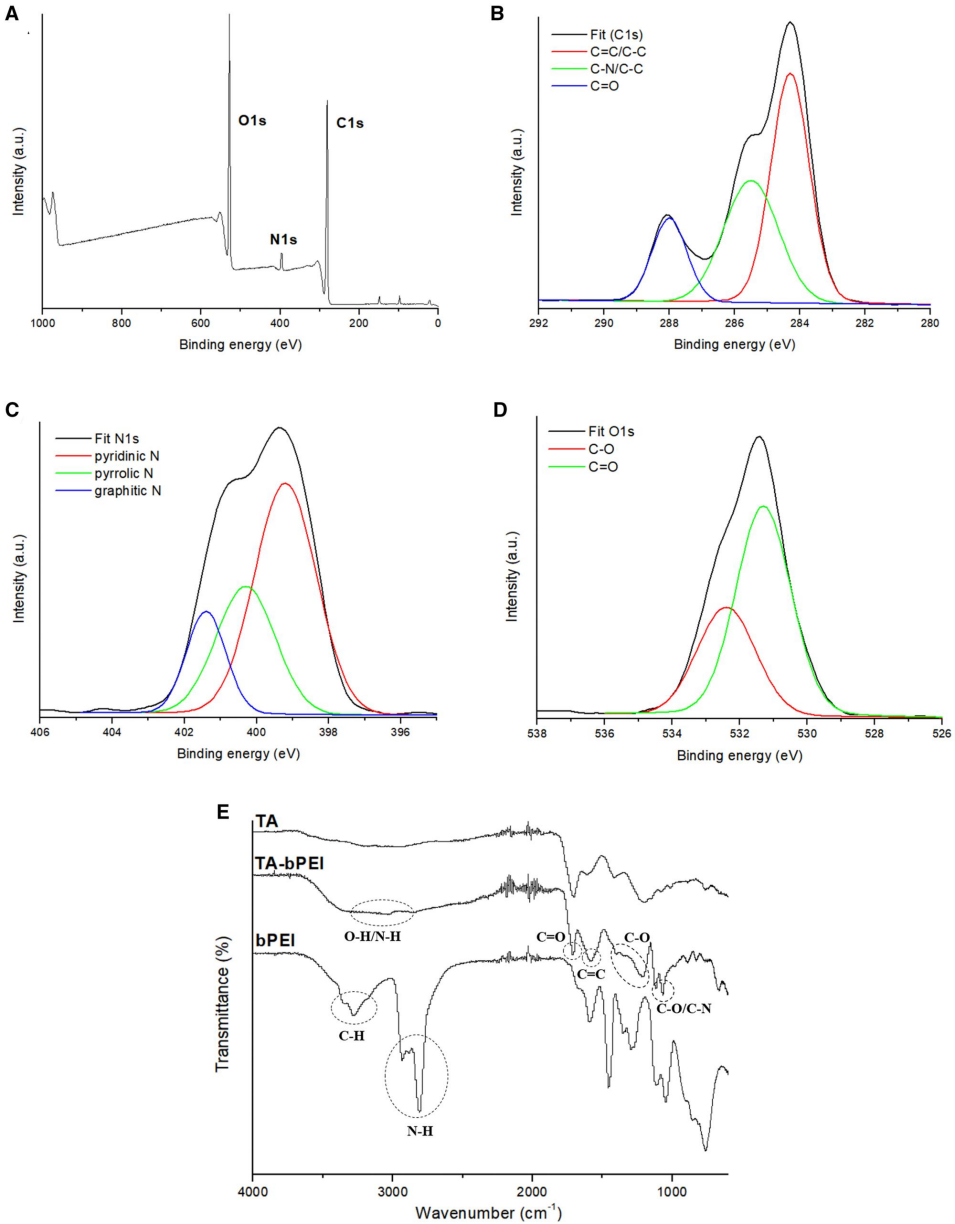
Chemical composition and surface functional groups of TA-bPEI CDs. Survey XPS spectrum of TA-bPEI CDs (**A**), and XPS curve fitting of the C 1s (**B**), N 1s (**C**), O 1s (**D**) peaks. FT-IR spectra of TA, bPEI and TA-bPEI CDs (**E**).

The XPS spectra based on survey analysis indicated the existence of three major elements, C, N and O, on the surface of the TA-bPEI CDs. Three typical C, N and O peaks were observed at 285, 396 and 528 eV, respectively (Figure 2A). According to the deconvolution, C1s spectrum displayed three types carbon: C=O at 288.1 eV, C–N/C–O at 286.6 eV and C=C/C–C at 284.6 eV (Figure 2B). N1s spectrum displayed pyridinic N at 399.2 eV, pyrrolic N at 400.3 eV and graphitic N at 401.4 eV (Figure 2C). The O1s spectrum displayed two peaks: C–O (532.3 eV) and C=O (531.5 eV) (Figure 2D). The FT-IR spectra of the TA, bPEI and TA-bPEI CDs are shown collectively in one figure (Figure 2E). FT-IR spectra were obtained to identify and analyse the molecular compounds and their binding states. The TA-bPEI CDs have wide absorption peaks of O–H/N–H at 2800–3500 cm^−1^ owing to the OH and NH_2_ groups, which are abundant on the surfaces of TA and bPEI. The peak at 1708 cm^−1^ was due to the stretching C=O vibration, and the peak at 1581 cm^−1^ was ascribed to the C=C stretching vibration. Peaks at 1215–1072 cm^−1^ originated from TA and bPEI owing to the C–N stretching vibration. The obtained spectra of the TA-bPEI CDs confirmed the binding of bPEI to TA through microwave-assisted heating and explained why the resultant zeta potential was positive.

### XRD and UV–vis absorption of TA-bPEI CDs

**Figure 3.**
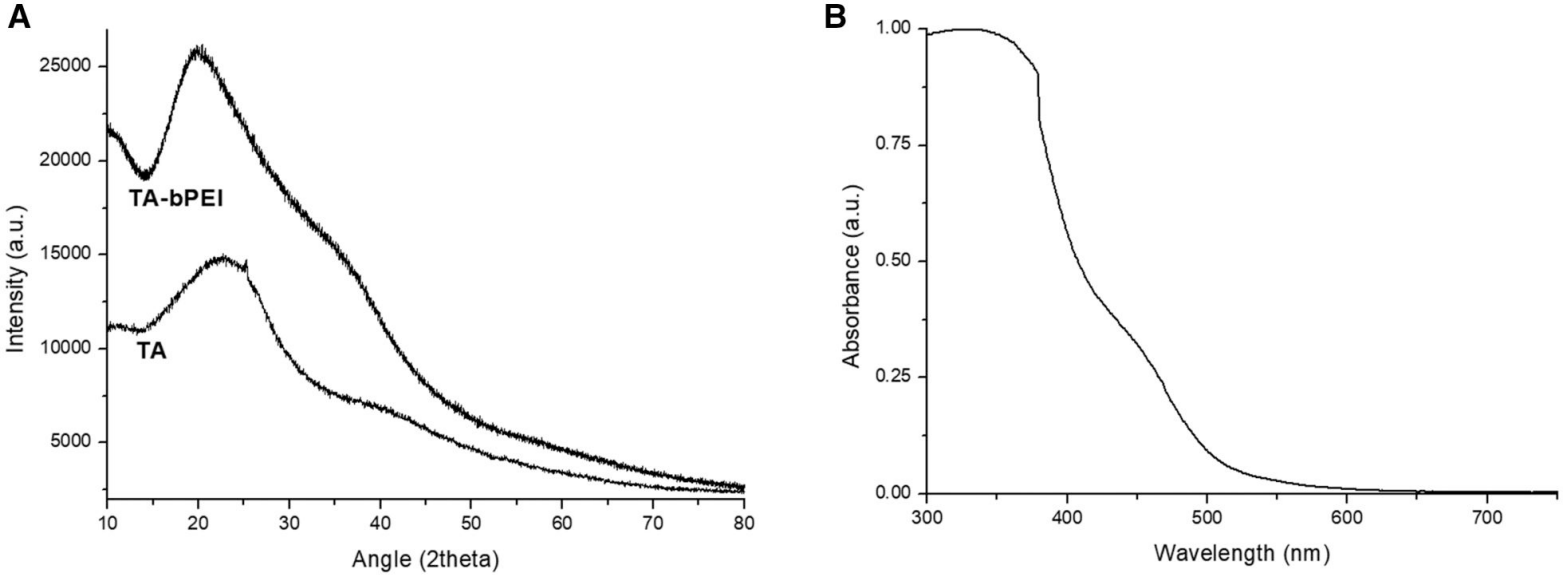
XRD Spectra of TA and TA-bPEI CDs. (**A**) They have one high and broad peak near 20°–23° and additional weak hump after 35°. (**B**) UV–vis absorption spectrum of TA-bPEI CDs.

The XRD pattern of the TA-bPEI CDs showed a broad peak at 20.4°, which corresponds to the (0 0 2) plane, and a weak amorphous hump near 36° (Figure 3A). The crystalline nature of graphite structure with (0 0 2) plane matches with a lattice spacing of approximately 0.32 nm as observed in HRTEM of TA-bPEI CDs. The XRD pattern of TA prepared in the furnace without bPEI showed a slightly shifted high peak at 22.9° and a weak hump near 41.9°. The UV–vis absorption spectra of the CDs are shown in Figure 3B. The TA-bPEI CDs exhibit an absorption peak near 349 nm and a weak hump centered near 450 nm. Above 500 nm, the curve decreased linearly to 650 nm. The absorption peak near 340 nm indicates the probable *n* → π* transitions of C=O bond from the surface groups.

### PL of TA-bPEI CDs

**Figure 4.**
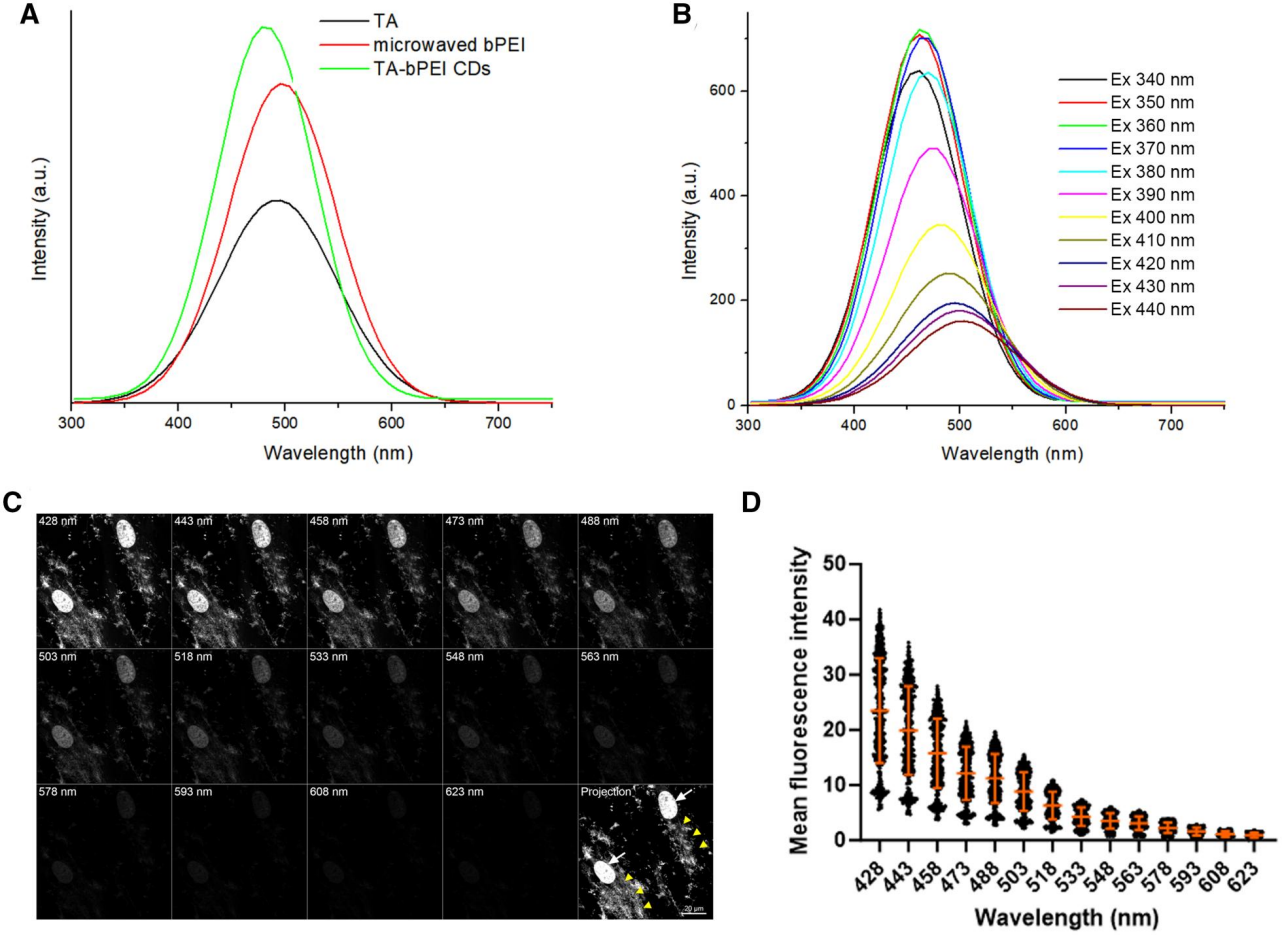
Photoluminescence and cellular imaging characteristics of TA-bPEI CDs. (**A**) Fluorescence (PL) spectra of TA and TA-bPEI CDs at 400 nm excitation. (**B**) Emission spectra of TA-bPEI CDs by excitation at 340–440 nm. The emission peak position and intensity were exponentially red shifted and decreased, respectively, as the excitation wavelength was gradually increased to 440 nm. (**C**) Series and projection of confocal microscopy images recorded at various wavelength ranges at 420–630 nm. The white arrow indicates a 4′,6-diamidino-2-phenylindole (DAPI)-positive nucleus, and the arrowhead indicates a TA-bPEI CDs-positive area. (**D**) Quantitative analysis of the mean fluorescence intensity of the TA-bPEI CDs-positive area.

The PL spectra of the tested materials are shown in Figure 4. They exhibited similar PL distributions and emission peaks at an excitation wavelength of 400 nm (Figure 4A). TA and microwaved bPEI exhibited peaks at approximately 487 nm. The TA-bPEI CDs, although exhibiting a slightly shifted peak position, have a similar PL distribution to those of TA and microwaved bPEI. The TA-bPEI CDs exhibit emission peaks at 464–506 nm upon excitation at 340–440 nm. The emission peak position shifts exponentially from 464 to 506 nm, and its peak intensity decreases exponentially after reaching a maximum upon excitation at 360 nm (Figure 4B) [25]. A lambda scan was conducted using a confocal microscopy to assess the physiological state of TA-bPEI CDs fluorescence at the single-cell level. Upon excitation at 405 nm, TA-bPEI CDs displayed a fluorescence peak at 428 nm within the cells (Figure 4C and D).

### Cytotoxicity of the TA-bPEI CDs

**Figure 5.**
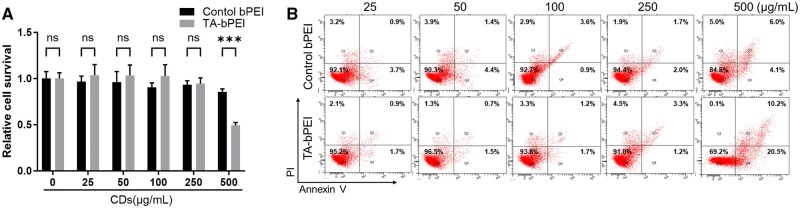
Cytotoxicity analysis of CDs using BMSCs *in vitro*. (**A**) CCK-8 assay of the CDs. (**B**) Flow cytometric analysis of the annexin V-FITC/PI staining in BMSCs. Data are shown as mean ± SD. ***P < 0.001, Student’s *t*-tests. n.s., not significant.

To assess the cytotoxicity of CDs on BMSCs, the cells were exposed to various concentrations (0, 25, 50, 100, 250 and 500 μg/ml) of control bPEI and TA-bPEI CDs for 48 h, and cell viability was determined using the CCK-8 assay. The results showed statistically insignificant decrease of BMSC viability after treatment of control bPEI up to 250 μg/ml. However, although not statistically significant, TA-bPEI-treated BMSC showed slightly greater viability than the control bPEI. Upon the treatment of 500 μg/ml, TA-bPEI-treated BMSC showed much less viability compared to that of control bPEI (Figure 5A). To further verify the cytotoxicity of CDs on BMSCs, an Annexin V/PI apoptosis assay was performed. Similar to the CCK-8 assay results, the population of apoptotic BMSCs (Annexin V+/PI− and Annexin V+/PI+) was greatly increased by treatment of 500 μg/ml of control bPEI and TA-bPEI treatment (Figure 5B).

### Osteogenic effects of TA-bPEI CDs

**Figure 6.**
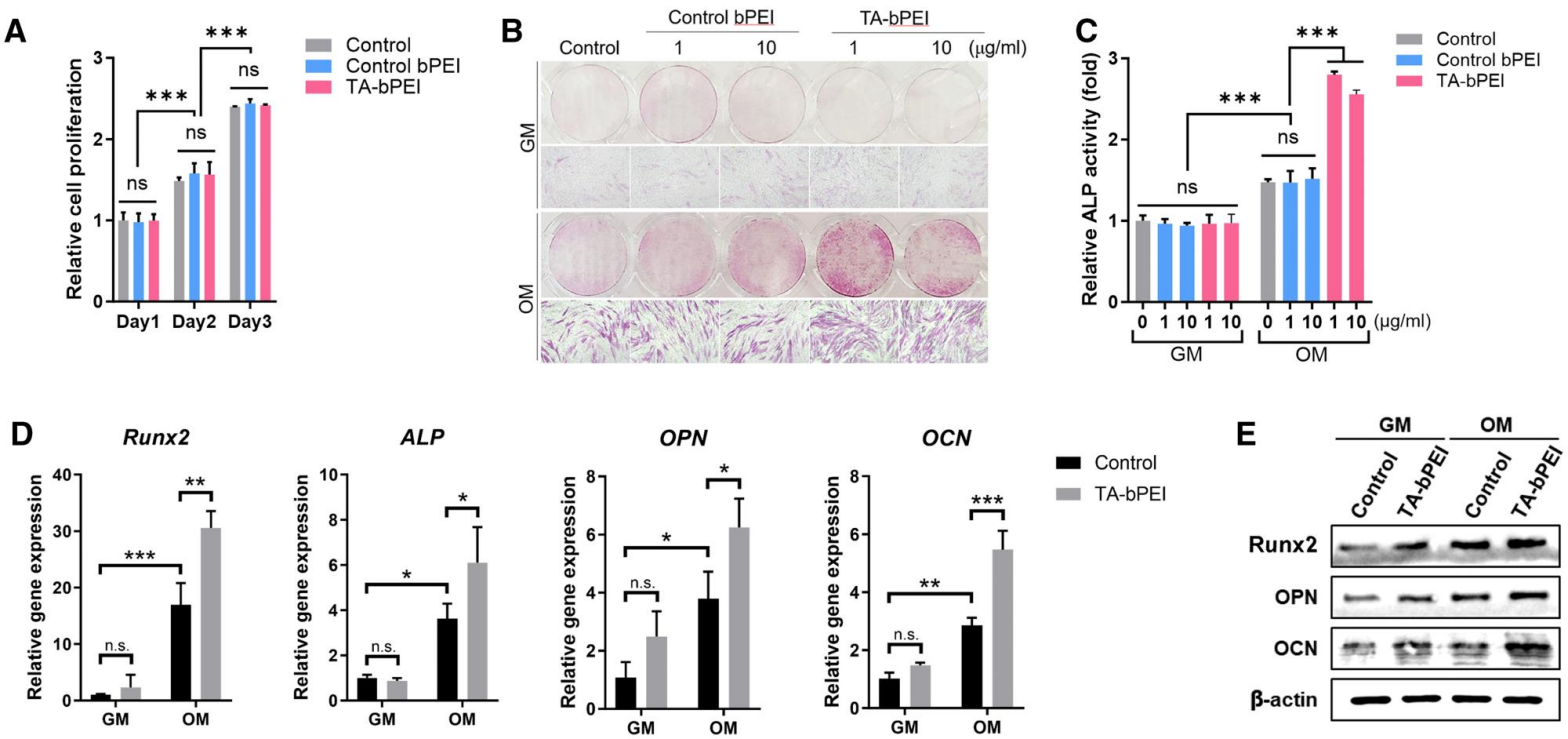
Effects of TA-bPEI CDs on proliferation and osteogenic differentiation of BMSCs. (**A**) The proliferation rate of BMSCs was assessed via a CCK-8 assay. (**B**) Representative images of ALP staining on day 4. (**C**) ALP activity was quantitatively evaluated. (**D**) mRNA expression of *Runx2*, *ALP*, *OPN* and *OCN* was analysed using qRT-PCR and normalized to β-actin expression. (**E**) Protein levels of Runx2, OPN and OCN were assessed by Western blotting, with β-actin used as the loading control. Data are shown as mean ± SD. *P < 0.05; **P < 0.01; ***P < 0.001, two-way ANOVA. n.s., not significant.

To examine if TA-bPEI CDs stimulate the proliferation of BMSCs, cells were exposed to 1 μg/ml CDs of control bPEI or TA-bPEI CDs for 3 days, and their proliferation was determined using the CCK-8 assay. Over time, the cells proliferated significantly. However, no differences in cell proliferation were observed between the test conditions (Figure 6A). BMSCs were cultured in growth medium (GM) and osteogenic medium (OM) for 4 days with CDs of two different concentrations, 1 and 10 μg/ml, and ALP activity was evaluated through ALP staining. The results revealed that adding CDs at concentrations of 1 and 10 μg/ml to the OM led to a significant enhancement in both the proportion of ALP-positive BMSCs and their ALP activity (Figure 6B and C). Additional studies confirmed that TA-bPEI treatment did not induce adipogenic or chondrogenic differentiation in BMSCs, as evidenced by the absence of notable alterations in the expression of adipogenic markers (*CEBPA****,*** *PPARA*; Supplementary Figure S1A) and chondrogenic markers (*Sox9, Col2a, ACAN*; Supplementary Figure S1B). Furthermore, experiments revealed that TA alone, even at equivalent concentrations, failed to induce osteogenic differentiation (Supplementary Figure S2A and B). Subsequent RT-qPCR analysis confirmed that the supplemented 1 μg/ml TA-bPEI CDs in OM further induced the expression of osteogenic-specific genes, such as *Runx2*, *ALP*, *OPN* and *OCN* (Figure 6D). Additionally, western blotting results showed that the expression of osteogenesis-specific proteins, such as RUNX2, OPN and OCN, was potentiated by treatment with TA-bPEI CDs during osteoblast differentiation of BMSCs (Figure 6E). These results strongly suggested that TA-bPEI facilitated the osteogenic differentiation of BMSCs.

### Effects of TA-bPEI CDs on *in vivo* bone regeneration

**Figure 7.**
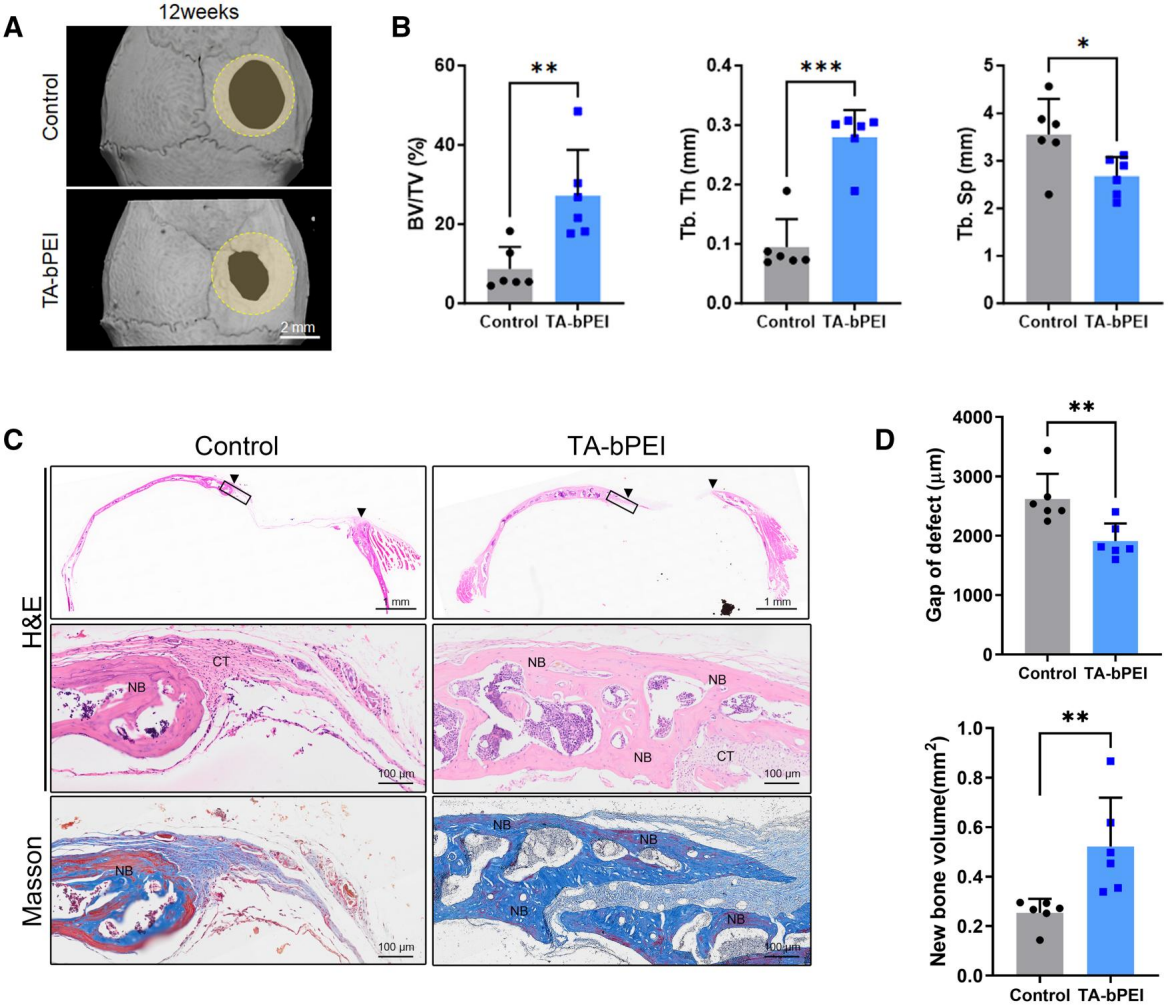
Effects of TA-bPEI CDs on bone repair in the calvarial defect model *in vivo*. (**A**) Representative images of micro-CT 3D reconstructions of calvarial defects of mice. Circles indicate original defect area. (**B**) Quantitative analysis of the ratio of tissue volume within the defected area (BV/TV), trabecular thickness (Tb.Th) and trabecular separation (Tb.Sp). (**C**) Representative histological images of calvarial defects stained with hematoxylin and eosin (H&E) and Masson’s trichrome. Higher magnification views of the outlined regions in the upper panels are shown in the lower panels. Arrowheads indicate the edges of the defect. (**D**) The gap distance of the calvarial defect was measured from H&E-stained images, while new bone volume was assessed using Masson’s trichrome-stained images. Data are shown as mean ± SD. (*n* = 6) *P < 0.05; **P < 0.01; ***P < 0.001, Student’s *t*-tests. CT, connective tissues; NB, new bone.

To investigate whether the TA-bPEI CDs could accelerate bone repair via bone regeneration, an *in vivo* bone defect model was established using mouse calvaria. Micro-CT examinations were performed 12 weeks after the placement of the CD-containing gel into the bone defect on the mouse calvaria. According to the micro-CT images, NB formation was limited in the control group, whereas the TA-bPEI CDs-treated group exhibited substantial NB growth along the defect margins (Figure 7A). Quantitative assessment of the micro-CT images revealed that the TA-bPEI CDs gel significantly enhanced bone morphometric parameters, increasing BV/TV and Tb.Th by 3.13- and 2.94-fold, respectively, relative to the control. Additionally, Tb.Sp in TA-bPEI CDs-treated defects was significantly reduced compared with control defects (Figure 7B). To evaluate whether the osteogenic effects of TA-bPEI CDs were associated with immune cell activity, we analysed immune cell infiltration at the early stage post-surgery. Immunofluorescence staining showed increased CD3-positive T cells and CD68-positive macrophages in all surgical groups compared to the WT control. However, there were no statistically significant differences between the control bPEI and the TA-bPEI-treated groups (Supplementary Figure S3A and B), suggesting the enhanced bone regeneration observed with TA-bPEI CDs may be attributed to their direct stimulation of MSC osteogenic differentiation rather than immune cell-mediated effects. Masson’s trichrome and hematoxylin and eosin (H&E) staining were performed on calvarial bone defects to assess tissue regeneration following the application of TA-bPEI CDs-based gel. H&E staining revealed increased NB formation in the TA-bPEI-treated group compared to the control, with well-organized connective tissue (CT) surrounding the defect site. Masson’s trichrome staining confirmed a significant increase in mineralized bone in the treated group. Quantitative analysis showed a substantial reduction in defect gap distance and a significant increase in NB volume in the TA-bPEI group compared to the control (Figure 7C and D). In summary, these findings indicate that TA-bPEI CDs significantly enhance bone regeneration by directly promoting osteogenic differentiation.

## Discussion

NB formation is a key aspect of complex physiological processes involved in bone healing in injured areas [26]. During this process, chemotactic stem cells are recruited to the site of injury and undergo osteoblastic differentiation, which contributes to the secretion of various bone matrix proteins. Following mineral deposition, bone tissue begins to regenerate, thereby resulting in the formation of NB to repair defects [27]. In this study, we demonstrated that TA-bPEI CDs effectively regulated osteogenic activity both *in vitro* and *in vivo*. As a novel active nanomaterial, TA-bPEI CDs diverge from conventional osteogenic stimulants and offer an alternative avenue for bone regeneration.

Cytotoxicity remains a major factor to be addressed when evaluating the feasibility of the TA-bPEI CDs for future clinical use. Upon treatment to 500 μg/ml of TA-bPEI CDs, the CCK-8 assay demonstrated a negligible decrease of BMSC viability up to 250 μg/ml, followed by a significant decrease at 500 μg/ml compared to that of control bPEI. Similarly, the flow cytometry-based apoptosis assay also provided evidence that treatment of TA-bPEI CDs significantly induces apoptosis only at a concentration of 500 μg/ml. The presence of a lower concentration of TA-bPEI CDs (1–10 μg/ml) substantially stimulated osteogenic differentiation in BMSCs without affecting cell proliferation. Several osteogenesis-related genes and proteins, such as Runx2, ALP, OCN and OPN, are involved in osteogenic differentiation [28]. During this process, the treated TA-bPEI CDs remarkably increased the expression of these osteogenic markers compared to the control bPEI. Runx2 plays a critical role in driving MSC commitment toward the osteoblast lineage and exerts significant control during the initial phases of osteoblast differentiation; however, it must be downregulated to facilitate subsequent bone maturation [29, 30]. ALP expression occurs early during osteoblast differentiation, diminishes as development proceeds and plays a significant role in mineral formation in bone tissues. Conversely, other genes such as OCN and OPN are significantly upregulated in mature osteoblast [31]. Additionally, our *in vivo* analysis of immune cell activity indicated that the TA-bPEI CDs did not significantly alter the recruitment or activation of CD3-positive T cells and CD68-positive macrophages compared to the control bPEI. This suggests that the enhanced bone regeneration observed with TA-bPEI CDs is likely driven by their direct stimulation of MSC osteogenic differentiation rather than through immune cell-mediated mechanisms. This is consistent with our *in vitro* findings, where TA-bPEI CDs demonstrated robust osteogenic potential by upregulating osteogenesis-related markers in BMSCs. In our study, treatment with TA-bPEI CDs induced the expression of a broad spectrum of osteogenic markers, ranging from those associated with early stages to those associated with later stages. This dual insight, which includes the lack of immune-mediated effects and the direct stimulation of MSC differentiation, highlights the robust potential of TA-bPEI CDs for promoting osteogenic activity through direct mechanisms.

TA (C_4_H_6_O_6_) is abundant in fruits, such as grapes and bananas. It has a negative potential due to its hydroxyl and carboxyl groups [32]. PEI [(C_2_H_5_N)_*n*_] is a polycationic polymer with a high positive charge density potential that is attributed to the repeating units of the protonatable amino nitrogen. The cationic nature of bPEI changed the zeta potential of TA-bPEI to positive after binding to the negatively charged TA. The resultant TA-bPEI CDs of high positive zeta potential value (27.5 ± 3.75 mV) is beneficial for interaction with negatively charged cells via electrostatic attraction. After treatment and time elapse, the aggregated and invaginated CDs on the cell membrane are internalized into cells via the endocytic pathway [15, 33, 34]. They can also be internalized via passive transport without invagination or vesicle encapsulation. Internalized CDs form endosomes within cells. CDs in endolysosomal compartments are released by bursting or leaking through membrane holes. CDs are then distributed to intracellular organs such as the nucleus, mitochondria and endoplasmic reticulum. An extremely small particle size (up to 10 nm) facilitated this process. Internalized TA-bPEI CDs affected the osteogenic differentiation of BMSCs by positively upregulating the activity of osteogenic markers, such as RUNX2, ALP, OCN and OPN, with significantly increased gene expression. Consistent with this mechanism, our results demonstrated that TA alone, even at equivalent concentrations, did not induce osteogenic differentiation. However, when TA was synthesized into TA-bPEI CDs, it effectively promoted osteogenic differentiation, as evidenced by enhanced ALP activity. These findings reinforce the critical role of the CD structure in facilitating cellular delivery and enhancing the osteogenic potential of TA. The resulting bone metabolism enhanced NB formation and decreased the defect gap, increasing the clinical potential for the application of CDs in bone regeneration or defect repair. In general, the distinct characteristics of nanoparticles affect their uptake by cells and subsequent intracellular routing, ultimately determining their fate and therapeutic efficacy. Therefore, further detailed case-dependent investigations are necessary.

The utilization of growth factors for tissue regeneration presents the challenge of delivery to injured tissues, while striving to develop minimally invasive detection methods. The use of CD-based approaches has demonstrated growing relevance in the field of bone regeneration. Gogoi *et al.* employed a carbodiimide coupling method to covalently functionalize peptides onto carbon dots, which were subsequently used for bone-tissue regeneration [35]. In addition, carbon dots co-doped with calcium and phosphorus facilitate osteogenic differentiation to repair calvarial defects *in situ* [36]. Consistent with these findings, our investigation demonstrated that the introduction of TA-based carbon dots elicited NB formation in a mouse calvarial defect model. Recently, the combination of nanoparticles with bulk bone minerals, such as hydroxyapatite and tricalcium phosphate (TCP), has become a common approach to improve their osteogenic capabilities for bone defect repair [37, 38]. However in the present study, CDs were synthesized using a one-step microwave process. Unlike other methods that incorporate functional materials or metal ions into carrier CDs, TA-bPEI CDs were synthesized using a simple one-pot process; thus, they were less sensitive to the synthesis process and consistent in their outcomes. Positive surface charges were facilitated by bPEI, although this polymer increased cellular toxicity, which is known to depend on the molecular weight and cellular type [39, 40].

In the present study, cell toxicity was tested up to 500 μg/ml concentration and resultant viability of BMSCs remained unaffected till 250 μg/ml. This value is much higher than that of the tested maximum concentration (10 μg/ml), and thus, it seems to evoke no cellular toxicity. Enhanced cell differentiation resulted in increased NB generation, which was evident from induced osteogenic genes and proteins and decreased gap distance in defects. Increased bone volume at the defect site would be beneficial in that it can reduce repair time. In clinics, repair time is directly related to hospitalization and hospital costs. The positive outcomes of the present study using TA-bPEI CDs may demonstrate their potential benefits for bone treatment.

## Conclusion

In this study, bPEI-attached TA-based CDs were synthesized and applied to BMSCs *in vitro* and *in vivo* in mouse calvarial defects. The TA-bPEI CDs have a high positive zeta potential, which enhances the attachment of CDs to the cell surface via electrostatic attraction. Treated TA-bPEI CDs did not affect cell viability at concentrations up to 250 μg/ml and similar cell apoptosis was confirmed via flow cytometry. BMSCs showed apparent cell differentiation by TA-bPEI CDs at a concentration of 1 μg/ml compared to the control bPEI. Enhanced cell differentiation was accompanied by the further induction of osteogenic gene and protein expression. As a result, NB filled over 62% of the initial calvarial bone defects with much reduced gap distance only by TA-bPEI CDs at a concentration of 1 μg/ml. The present study suggests that low concentrations of TA-bPEI CDs have great potential for bone regeneration and repair in clinical practice.

## Supplementary Material

rbaf030_Supplementary_Data

## Data Availability

The datasets generated and/or analysed during the current study are not publicly available but are available from the corresponding author on reasonable request.
