# Supplementary material for: Tartaric acid-branched polyethyleneimine carbon dots promote repair of bone defect via osteogenic differentiation
Source: Regen Biomater. 2025 May 16;12:rbaf030. doi: 10.1093/rb/rbaf030 (PMC12098262; doi:10.1093/rb/rbaf030)
Supplement: rbaf030_Supplementary_Data [file rbaf030_supplementary_data.zip]

**Supplementary Information:**

Tartaric acid-branched polyethyleneimine carbon dots promote repair of bone defect via osteogenic differentiation

Soon Chul Heo^a,b^, Hae Won Shin^c^, Dong Joon Lee^d^, Franklin Garcia-Godoy^e^, Bo Ram Keum^a^, Yong Hoon Kwon^f*^, Hyung Joon Kim^a*^

^a^Department of Oral Physiology, Periodontal Diseases Signaling Network Research Center, Dental and Life Science Institute, School of Dentistry, Pusan National University, Yangsan 50612, Korea

^b^Institute of Tissue Regeneration Engineering (ITREN), Mechanobiology Dental Medicine Research Center, Dankook University, Cheonan 31116, Republic of Korea.

^c^Department of Neurology, College of Medicine, University of Tennessee Health Science Center, Memphis, TN 38163, USA

^d^Oral and Craniofacial Health Sciences, Adams School of Dentistry, University of North Carolina at Chapel Hill, Chapel Hill, NC 27514, USA

^e^Department of Bioscience Research, College of Dentistry, University of Tennessee Health Science Center, Memphis, TN 38103, USA

^f^Department of Dental Materials, School of Dentistry, Pusan National University, Yangsan 50612, Korea

^*^Corresponding authors

E-mail addresses: y0k0916@pusan.ac.kr (Y.H.K.), hjoonkim@pusan.ac.kr (H.J.K.)

**Materials and methods**

**Adipogenic and Chondrogenic Differentiation**

For adipogenic differentiation, the medium (adipogenic medium, AM) consisted of α-MEM supplemented with 10% fetal bovine serum (FBS), 1% penicillin-streptomycin (P/S), 10 nM dexamethasone, 50 μg/mL L-ascorbic acid, and insulin-transferrin-selenium (Thermo Fisher Scientific). For chondrogenic differentiation, the medium (chondrogenic medium, CM) consisted of α-MEM supplemented with 1% P/S, 10 nM dexamethasone, 50 μg/mL L-ascorbic acid, insulin-transferrin-selenium, and 10 ng/mL recombinant human transforming growth factor-β1 (TGF-β1, PeproTech, Cranbury, NJ, USA). BMSCs were cultured in the respective differentiation media for 14 days. After the incubation period, total RNA was purified and analyzed using quantitative reverse transcription PCR (qRT-PCR). The primer sequences used were as follows: CEBPA, 5′-AGGAGGATGAAGCCAAGCAGCT-3’, 5′-AGTGCGCGATCTGGAACTGCAG-3′; PPARA, 5′-AGCCTGCGAAAGCCTTTTGGTG-3′, 5′-GGCTTCACATTCAGCAAACCTGG-3′; Sox9, 5′-AGGAAGCTCGCGGACCAGTAC-3′, 5′-GGTGGTCCTTCTTGTGCTGCAC-3′; Col2a, 5′-GGCAATAGCAGGTTCACGTACA-3′, 5′-CGATAACAGTCTTGCCCCACTT-3′; ACAN, 5′-ACTTCCGCTGGTCAGATGGA-3′, 5′-TCTCGTGCCAGATCATCACC-3′.

**Immune Cell Analysis in a Mouse Calvarial Defect Model**

Mice were divided into three groups: wild-type (WT, no surgery), control bPEI-treated, and TA-bPEI-treated. For the surgical groups, a 4-mm defect was created in the calvarial bone using a dental bur, followed by the application of either control bPEI or TA-bPEI mixed with Matrigel to the defect site. Tissues were collected three days post-surgery, fixed in 4% paraformaldehyde, decalcified, and cryosectioned for further analysis. Immunofluorescence staining was performed on cryosections using primary antibodies against CD3 and CD68 (BioLegend, San Diego, CA, USA). Donkey anti-rat Alexa Fluor 647 (Invitrogen) was used as the secondary antibody, and nuclei were counterstained with DAPI. Images of the defect regions were captured using confocal microscopy (K1-fluo; Nanoscope Systems, Daejon, Korea), and CD3- or CD68-positive cells were quantified using ImageJ. Positive cells were analyzed as a percentage of DAPI-positive nuclei per high-power field (HPF).


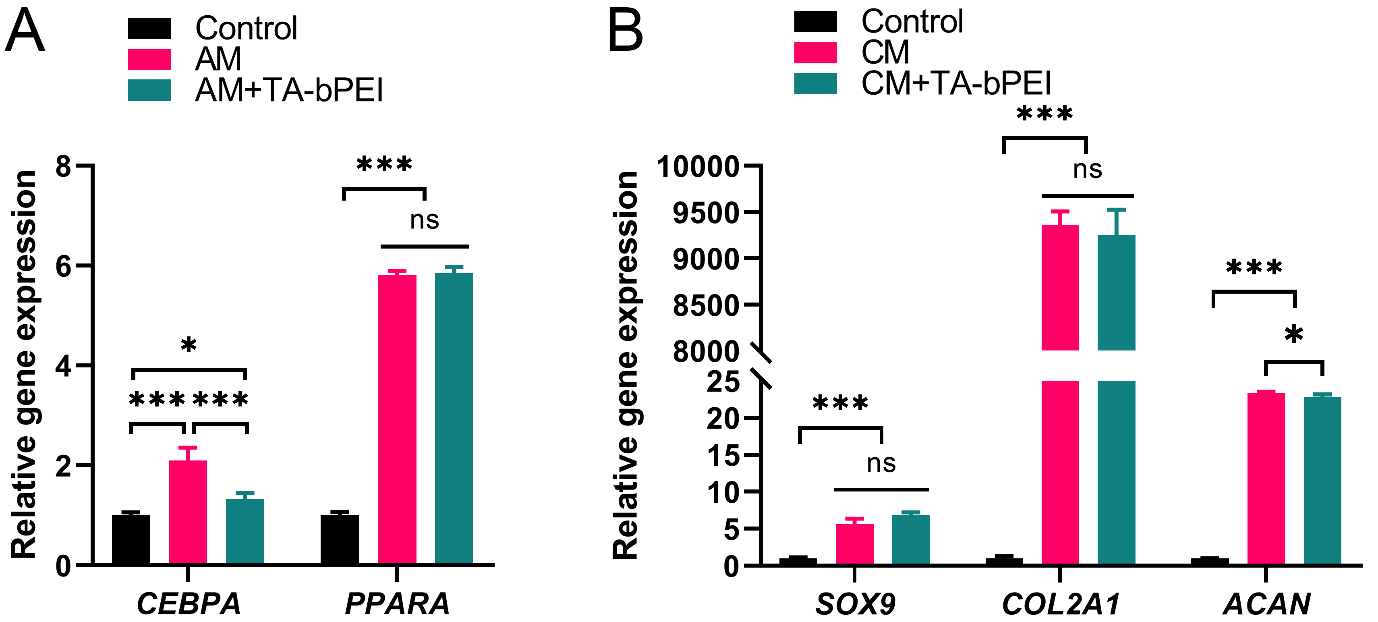


Supplementary Figure 1. Effects of TA-bPEI on adipogenic and chondrogenic differentiation of BMSCs. (A) Adipogenic differentiation was induced by culturing BMSCs in adipogenic medium (AM) or AM supplemented with TA-bPEI (AM+TA-bPEI) for 14 days. Gene expression of adipogenic markers *CEBPA* and *PPARA* was analyzed by qRT-PCR. (B) Chondrogenic differentiation was induced by culturing BMSCs in chondrogenic medium (CM) or CM supplemented with TA-bPEI (CM+TA-bPEI) for 14 days. Gene expression of chondrogenic markers *SOX9*, *COL2A1*, and *ACAN* was analyzed by qRT-PCR. Data are shown as mean ± SD. **P* < 0.05; ***P* < 0.01; ****P* < 0.001, one-way ANOVA. n.s., not significant.


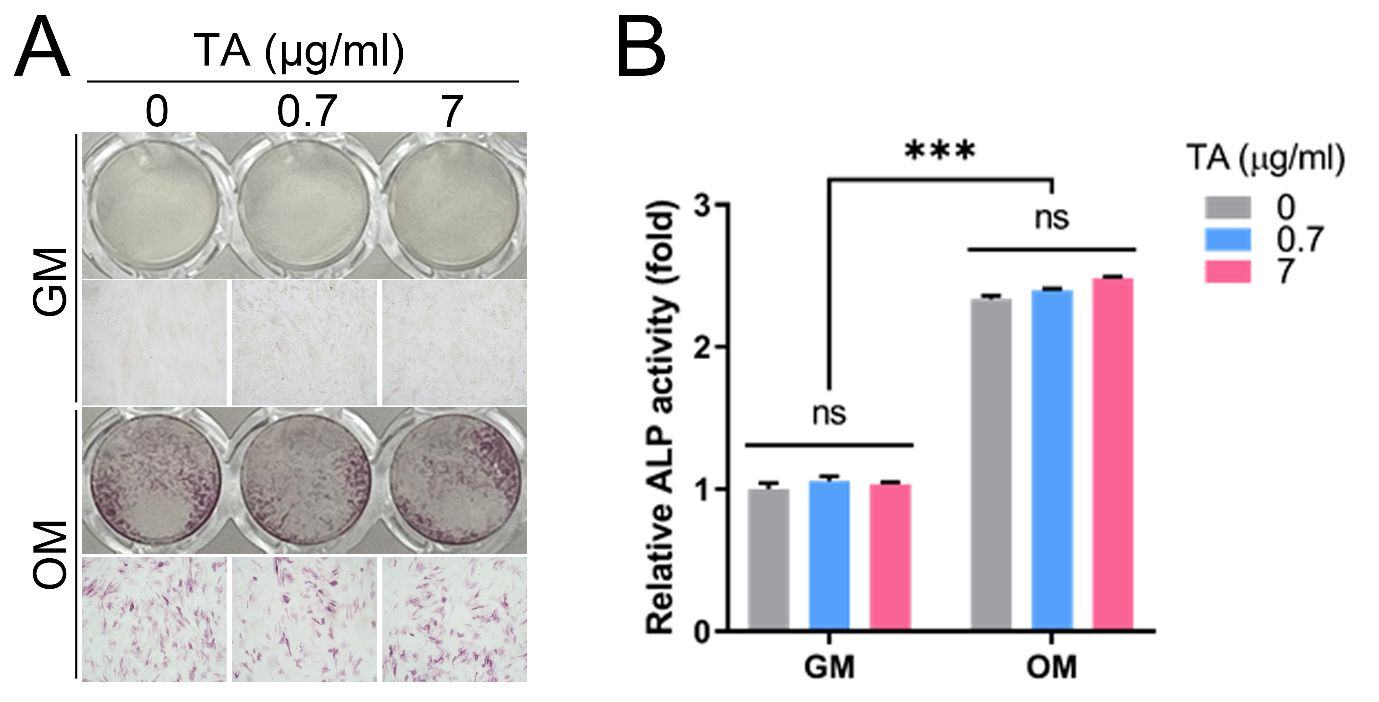


Supplementary Figure 2. Effects of TA alone on osteoblast differentiation in BMSCs. (A) Alkaline phosphatase (ALP) staining of BMSCs cultured in growth medium (GM) or osteogenic medium (OM) supplemented with 0, 0.7, or 7 μg/mL of tartaric acid (TA) for 4 days. (B) Quantitative analysis of ALP activity. Data are shown as mean ± SD. ****P* < 0.001, two-way ANOVA. n.s., not significant.


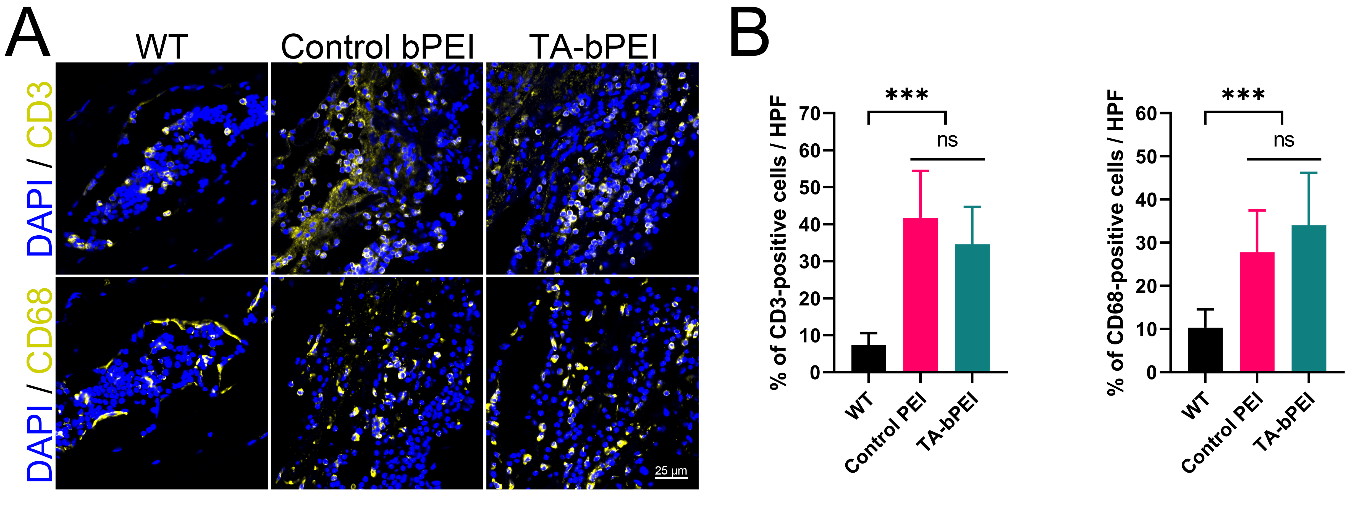


Supplementary Figure 3. Effects of TA-bPEI CDs on immune cell infiltration in a mouse calvarial defect model. (A) Representative immunofluorescence images of CD3-positive T cells (top, yellow) and CD68-positive macrophages (bottom, yellow) in wild type (WT) mice or in the defect regions of control bPEI-treated, and TA-bPEI-treated groups at 3 days post-surgery. Cell nuclei were counterstained with DAPI (blue). Scale bar: 25 μm. (B) Quantitative analysis of the percentage of CD3-positive (left) and CD68-positive (right) cells per high-power field (HPF). Data are shown as mean ± SD. ****P* < 0.001, one-way ANOVA. n.s., not significant.
